# Supplementary material for: A model for designing intraocular pressure-regulating glaucoma implants
Source: PLoS One. 2022 Sep 2;17(9):e0273672. doi: 10.1371/journal.pone.0273672 (PMC9439203; doi:10.1371/journal.pone.0273672)
Supplement: Attachment — Submitted filename: Report on PONE-D-22-04970.pdf [file pone.0273672.s002.pdf]

Report on

A computational model for designing intraocular pressure regulating glaucoma implants

by

Inês C.F. Pereira, Hans M. Wyss, Leonard Pinchuk, Henny J.M. Beckers, Jaap M.J. den Toonder

Glaucoma is the damage of the optical nerve caused by a high intraocular pressure (IOP). The different types of treatment (drug therapy, laser, surgical) aim at lowering IOP. The paper addresses the problem of tuning the properties of a glaucoma drainage device surgically implanted- namely the hydraulic resistance-in order to prevent ulterior hypotony (IOP excessive lowering) or the increase of IOP due to bleb scarring.

The manuscript presents two complementary approaches: a mathematical model of aqueous humor drainage, based on a Coupled Navier Stokes+Darcy model, and a laboratorial model based on a prototype where microfluidic experiments are performed.

The manuscript is interesting and it provides insights into the design of future glaucoma implants. However, we consider that a certain number of questions should be clarified:

1) The authors model the flow in the **shunt** with **Navier-Stokes** equation and the permeation through the **bleb and the conjunctiva** with **Darcy** equation. Nonetheless, the physiological properties of the bleb suggest that modelling the flow there with Navier-Stokes equation is more appropriate and could lead to results that are more rigorous. Arguments explaining the rationale under the authors modelling choice in the bleb should be presented.
2) Regarding numerical simulations with Comsol Multiphysics, no details are discussed namely the need for local mesh refinements (interface shunt/bleb/scar tissue?).

3) The model accounts for shunt geometry before application, but not to its deformation (curvature) after insertion.
4) As stated in the paper the aqueous humor production varies with the circadian rhythm. The authors present their simulations with an aqueous humor production Q_{in}=2,5 υl/min. Wouldn't using a weighted average of AH production (during 24 hours) have led to a more realistic model?
5) The authors mention that they essentially follow the model in Gardiner [15]. The originality of the contribution of the present manuscript should be clearly stated.
