# Supplementary material for: A model for designing intraocular pressure-regulating glaucoma implants
Source: PLoS One. 2022 Sep 2;17(9):e0273672. doi: 10.1371/journal.pone.0273672 (PMC9439203; doi:10.1371/journal.pone.0273672)
Supplement: Attachment — Submitted filename: Rebuttal letter.docx [file pone.0273672.s003.docx]

**Response to reviewers**

We thank the reviewers for their thoughtful and encouraging remarks. We also appreciate their critical and useful comments that have led us to incorporate meaningful revisions in our manuscript. In the following text, we specifically address the points of the reviewers with our response in red. The corresponding changes are highlighted in yellow in the revised manuscript.

**Reviewer #1:**

**Question 1 –** What are the main differences between the mathematical model presented here and the one included in the reference [15]?

The main difference between the mathematical model presented in this manuscript and the one described by Gardiner and co-workers is that, for simplification, we did not consider the outflow of aqueous humor through the natural outflow pathways (trabecular and non-trabecular/uveoscleral) in our simulations. The reason for this is that, in a glaucomatous eye and when a glaucoma drainage device is implanted, most aqueous humor drains through the glaucoma implant into the bleb, which represents the easiest fluidic pathway [1]. It is hypothesized in the literature that only 10% of the aqueous humor drains through the natural outflow pathways after trabeculectomy is performed [2]. This information was indeed missing, so we have added it in the revised manuscript in the section Methods, subsection “Governing equations” (lines 185-192).

Additionally, the values attributed to the parameters used for the calculation of the bleb pressure in the numerical simulations were different from the values used by Gardiner and co-workers. The values that we used were chosen after an extensive literature research has been made, and therefore we consider them more accurate than the ones presented in reference [15]. We have also used the PRESERFLO MicroShunt dimensions (70 µm of inner diameter) to represent the glaucoma drainage device in our model, whereas Gardiner and co-workers used the Molteno3 dimensions, with a tube inner diameter of 200 µm.

**Question 2 –** How are coupled the equations for the fluid in the shunt domain with Darcy’s equation in the bleb?

To couple the equations for the fluid in the shunt domain with Darcy’s equation in the bleb we have attributed the pressure calculated in the bleb at the outlet boundary condition of the tube, and applied the normal inflow velocity of the fluid leaving the tube at the inlet boundary condition of the bleb. This information was added in the revised manuscript in section Methods, subsection “Governing equations” (lines 202-205).

**Question 3 –** The interface and the boundary conditions should be written mathematically.

We thank the reviewer for the valuable suggestion of writing mathematically the interface and boundary conditions in the manuscript. We have summarized this information in a table and added it as Table 2 in the revised manuscript in section Methods, subsection “Governing equations” (lines 262-267).

**Question 4 –** The main objective of the paper is to present a computational model. It should be pointed out that the computational model is not the mathematical model presented in the paper. In fact, to define a computational model is necessary to apply numerical methods to the partial differential equations. These methods allow the replacement of the continuous problem by a set of discrete equations that can be used to develop a software package. In the paper we do not have any reference to the computational methods used to approximate the system of partial differential equations and their interface and boundary conditions.

We agree with the reviewer that the model we present strictly is not a computational model; we indeed describe the mathematical model which is numerically solved using the commercial FEM package COMSOL without presenting the actual discrete equations implemented and solved by COMSOL. To avoid any misunderstanding, we replaced the wording “computational model” either by “mathematical model” or by the more neutral “model” throughout the manuscript, and we emphasize the use of COMSOL to numerically solve the model (in the abstract (lines 25-26), the methods section (line 119), and the concluding section (lines 539-540)).

**Question 5 –** In line 58, the authors state that the fluid in the bleb can be absorbed by the subconjunctival or episcleral vasculature. This fact means that the fluid can be absorbed by the sclera and go to these blood vessels? Why this fact is not included in the mathematical model?

Indeed, it is generally reported that the aqueous humor flowing into the bleb after glaucoma filtration surgery is reabsorbed into the circulation by the subconjunctival or episcleral vasculature. However, tracer-based studies used to further investigate the subconjunctival outflow indicate that the conjunctival blood vessels and conjunctival lymphatic system represent the main structures involved in removing the excess interstitial subconjunctival fluid (aqueous humor), thereby reducing the pressure in the eye [1,3–5]. Taking this into account, we have decided to not include the absorption of aqueous humor by the episcleral vasculature in our model and only consider the conjunctival route of absorption. The above arguments explaining our decision were added in the revised manuscript in section Methods, subsection “Geometry” (lines 128-135).

**Question 6 –** The bleb is a space filled with aqueous humour. The authors use Darcy’s law in description of the aqueous humour dynamics. This fact means that the bleb is seen as a porous medium. This is the right approach? Why the Navier-Stokes equations are not used?

As postulated by Gardiner and co-workers, most biological tissues may be conceived as a two-phase porous media comprised of liquid and solid phases: the extracellular fluid being the liquid phase, and the extracellular matrix and cytoskeleton of cells being the solid phase. As such, a functioning filtering bleb can be also seen as a porous medium since it has numerous microcysts (clear spaces) filled with aqueous humor, lined by epithelium containing goblet cells, and surrounded by a superficial stroma that is composed of loosely arranged connective tissue of irregular collagen with a slight or absent subepithelial inflammatory response of lymphocytes, macrophages, and fibroblasts [4,6–10]. Additionally, the early phase of conjunctival wound healing following glaucoma filtration surgery involves leakage of plasma proteins and blood cells (solid material) from the disrupted vasculature into the bleb site (filled with fluid) [11]. Considering the above-mentioned arguments, we have decided to adopt Darcy’s law for flow in porous media to describe the aqueous humor dynamics in the bleb. These arguments were also included in the revised manuscript in section Methods, subsection “Governing equations” (lines 197-202).

**Reviewer #2:**

**Question 1 –** The authors model the flow in the shunt with Navier-Stokes equation and the permeation through the bleb and the conjunctiva with Darcy equation. Nonetheless, the physiological properties of the bleb suggest that modelling the flow there with Navier-Stokes equation is more appropriate and could lead to results that are more rigorous. Arguments explaining the rationale under the authors modelling choice in the bleb should be presented.

The arguments explaining our modelling choice in the bleb can be found in the above answer to Question 6 from Reviewer #1. We thank the reviewer for the valuable suggestion of explaining in the manuscript the rationale of modelling the fluid flow though the bleb using the Darcy’s law for porous medium. We have added this information in the revised manuscript in section Methods, subsection “Governing equations” (lines 197-202).

**Question 2 –** Regarding numerical simulations with Comsol Multiphysics, no details are discussed namely the need for local mesh refinements (interface shunt/bleb/scar tissue?).

In COMSOL Multiphysics, a user-controlled mesh was used where the maximum element size was set to 0.005 mm. Local mesh refinements were not needed, since the average quality of the mesh was 95.98 %, and the minimum quality obtained was 55.59 %. This information was indeed missing in the manuscript, and was added in section Methods (lines 120-122).

Below some zoomed portions of the mesh quality plot generated in COMSOL are shown, with the scale bar indicating the quality of the mesh from 0 to 1 (being 1 the best possible mesh quality).

**Fig. 1.** Plot of the mesh quality at the inlet of the shunt domain.

**Fig. 2.** Plot of the mesh quality at the interface between the shunt domain and the bleb domain.

**Fig. 3.** Plot of the mesh quality in the subconjunctival tissue domain (most exterior part).

**Question 3 –** The model accounts for shunt geometry before application, but not to its deformation (curvature) after insertion.

A slight bend/curvature of the PRESERFLO Microshunt after implantation will not have a measurable effect on the flow rate. To the best of our knowledge, there are no studies reporting otherwise. From the opinion of one of the co-authors of this paper (H.J.M.B.), who is an ophthalmologist and glaucoma specialist with more that 23 years of experience in glaucoma surgery, current implants made of silicone or SIBS will not significantly bend or kink when placed into the eye. Bending has been indeed observed with the XEN-45 implant, impacting negatively the surgical outcome. This implant is very thin and is made of material comprised of porcine gelatin cross-linked with glutaraldehyde, which is extremely deformable as compared with silicone or SIBS. However in our manuscript we only consider the PRESERFLO Microshunt and we do not consider the XEN-45 implant.

**Question 4 –** As stated in the paper the aqueous humor production varies with the circadian rhythm. The authors present their simulations with an aqueous humor production Qin=2,5 l/min. Wouldn't using a weighted average of AH production (during 24 hours) have led to a more realistic model?

As mentioned in the manuscript, the aqueous humor production rate varies slightly between the waking and sleeping hours: 3 μL/min in the morning, 2.5 μL/min in the afternoon, and 1.5 μL/min during the night. The value of 2.5 μL/min was chosen as an approximated average value of aqueous humor production rate during a period of 24 hours. The choice of this value was also supported from the literature [12]. Reference [12] was indeed missing in the manuscript, and we have included it in section Methods, subsection “Governing equations” (lines 184-186).

**Question 5 –** The authors mention that they essentially follow the model in Gardiner [15]. The originality of the contribution of the present manuscript should be clearly stated.

The model presented in this manuscript describing the fluid removal from the eye through a glaucoma drainage device, its drainage into the filtering bleb and absorption by the subconjunctival vasculature, was indeed based on the work of Gardiner and co-workers. Gardiner and co-workers used this model to investigate how the IOP is influenced by some factors such as aqueous humor production and outflow, bleb geometry, subconjunctival tissue conductivity and tissue absorptive capacity. We, on the other hand, applied an adapted version of this model to study the possibility to **control** intraocular pressure in glaucoma patients by using a glaucoma implant with adjustable hydrodynamic resistance. To the best of our knowledge, this is the first time that such a numerical model was used to obtain insight into the adjustments in the implant’s hydrodynamic resistance that are required for IOP control when the two most common postoperative complications following glaucoma filtration surgery take place, hypotony and bleb scarring. The results reported in our manuscript may, for the first time, provide guidelines to support the design of future glaucoma implants with adjustable hydrodynamic resistances.

We are very grateful for this important suggestion of stating clearly in the manuscript the originality of our work. We have elaborated on this in section Methods (lines 109-117).

**References:**

1.	Lee JY, Heilweil G, Le P, Saraswathy S, Hong Y-K, Girkin CA, et al. Structural Confirmation of Lymphatic Outflow from Subconjunctival Blebs of Live Humans. Ophthalmol Sci. 2021;1(4):100080.

2.	Kotliar KE, Kozlova T V., Lanzl IM. Postoperative aqueous outflow in the human eye after glaucoma filtration surgery: biofluidmechanical considerations. Biomed Tech. 2009;54(1):14–22.

3.	Yu DY, Morgan WH, Sun X, Su EN, Cringle SJ, Yu PK, et al. The critical role of the conjunctiva in glaucoma filtration surgery. Prog Retin Eye Res. 2009;28(5):303–28.

4.	Lee JY, Akiyama G, Saraswathy S, Xie X, Pan X, Hong YK, et al. Aqueous humour outflow imaging: seeing is believing. Eye. 2021;35(1):202–15.

5.	Freitas-Neto CA, Costa RA, Kombo N, Freitas T, Oréfice JL, Oréfice F, et al. Subconjunctival indocyanine green identifies lymphatic vessels. JAMA Ophthalmol. 2015;133(1):102–4.

6.	Schlunck G, Meyer-ter-Vehn T, Klink T, Grehn F. Conjunctival fibrosis following filtering glaucoma surgery. Exp Eye Res. 2016;142:76–82.

7.	Mastropasqua R, Fasanella V, Brescia L, Oddone F, Mariotti C, Di Staso S, et al. In Vivo Confocal Imaging of the Conjunctiva as a Predictive Tool for the Glaucoma Filtration Surgery Outcome. Invest Ophthalmol Vis Sci. 2017;58(6):BIO114–20.

8.	Yılmaz SG, Değirmenci C, Palamar M, Yağcı A. Evaluation of filtering bleb function after trabeculectomy with mitomycin C using biomicroscopy, anterior segment optical coherence tomography and in vivo confocal microscopy. Turkish J Ophthalmol. 2015;45(4):132–7.

9.	Amar N, Labbé A, Hamard P, Dupas B, Baudouin C. Filtering Blebs and Aqueous Pathway. An Immunocytological and In Vivo Confocal Microscopy Study. Ophthalmology. 2008;115(7):1154–61.

10.	Guthoff R, Schlunck G, Klink T, Grehn F. Filtering Bleb Imaging with Confocal Laser Technology (Rostock Cornea Module). In: Grehn F, Stamper R, editors. Glaucoma. Germany: Springer-Verlag Berlin Heidelberg; 2006. p. 157–69.

11.	Khaw PT, Bouremel Y, Brocchini S, Henein C. The control of conjunctival fibrosis as a paradigm for the prevention of ocular fibrosis-related blindness. “Fibrosis has many friends.” Eye [Internet]. 2020;34(12):2163–74. Available from: http://dx.doi.org/10.1038/s41433-020-1031-9

12.	Thygesen J. Late Hypotony. In: Shaarawy TM, Sherwood MB, Crowston JG, editors. Glaucoma. 2nd ed. London, England, UK: Elsevier/Saunders; 2015. p. 863–81.
